# Spatio-Temporal Positron Emission Tomography Reconstruction with Attenuation and Motion Correction

**DOI:** 10.3390/jimaging9100231

**Published:** 2023-10-20

**Authors:** Enza Cece, Pierre Meyrat, Enza Torino, Olivier Verdier, Massimiliano Colarieti-Tosti

**Affiliations:** 1 Department of Biomedical Engineering and Health Systems, KTH Royal Institute of Technology, 10044 Stockholm, Sweden; enza.cece@unina.it (E.C.); meyrat@kth.se (P.M.); 2 Deptartment of Chemical Engineering, Materials and Production, University of Naples Federico II, 80131 Naples, Italy; enza.torino@unina.it; 3 Department of Computing, Mathematics, and Physics, HVL Western Norway University of Applied Sciences, 5063 Bergen, Norway; olivier.verdier@hvl.no; 4 Department of Clinical Science, Intervention & Technology, Karolinska Institutet, 171 77 Stockholm, Sweden

**Keywords:** PET, tomographic reconstruction, motion correction, attenuation correction, deep learning, MLAA

## Abstract

The detection of cancer lesions of a comparable size to that of the typical system resolution of modern scanners is a long-standing problem in Positron Emission Tomography. In this paper, the effect of composing an image-registering convolutional neural network with the modeling of the static data acquisition (i.e., the forward model) is investigated. Two algorithms for Positron Emission Tomography reconstruction with motion and attenuation correction are proposed and their performance is evaluated in the detectability of small pulmonary lesions. The evaluation is performed on synthetic data with respect to chosen figures of merit, visual inspection, and an ideal observer. The commonly used figures of merit—Peak Signal-to-Noise Ratio, Recovery Coefficient, and Signal Difference-to-Noise Ration—give inconclusive responses, whereas visual inspection and the Channelised Hotelling Observer suggest that the proposed algorithms outperform current clinical practice.

## 1. Introduction

PET (Positron Emission Tomography) is a medical imaging modality that reconstructs the 3D distribution of metabolic activity by detecting the photons emitted during the in vivo annihilation of free electrons with positrons from an injected radioactive tracer. In principle, cancer lesions should be visible in the reconstructed image with high contrast compared to the surrounding healthy tissue thanks to their particular metabolic fingerprints (e.g., higher sugar metabolism in fluorodeoxyglucose PET). However, the long acquisition time required to collect projection data with an acceptable noise level leads to motion artefacts that strongly affect lesions’ contrast-to-noise-ratio and, therefore, the diagnostic power of PET images. This is of particular relevance when trying to image lesions with sizes comparable to the system resolution and that are continuously moving around during data acquisition.

As an example, a current challenge in the PET diagnosis of lung cancer at an early stage is the visibility of lesions of dimensions of one centimetre or less, which will be displaced by a few times their dimension during the respiratory cycle.

One way of avoiding motion blurring is to divide projection data into a number of different data subsets (gates) in which the patient (and, therefore, the activity distribution) can be considered stationary. Gating can be achieved by means of an external gate-triggering device or by analysing the data itself. Once gated data is available, one can choose to reconstruct each gate separately or simply one of the gates (usually the one with more counts) avoiding, in this way, motion artefacts. The drawback with this approach is the higher noise level of the reconstructed images that can, especially for smaller lesions, result in scarce visibility and, consequently, uncertain diagnosis. For this reason, attempts have been made to jointly estimate the image and motion in order to reconstruct a single stationary image with the full statistics of the entire projection data set. On passing, please note that, in this work, only changes in the radiotracers distribution due to patient movement are considered and the kinetics of the radiotracer distribution during the acquisition is not taken into consideration. However, the two problems have many features in common and can be tackled using similar methods [1]. Similar ideas of minimising an objective function that depends on the parameters of both the image and of a prior motion model can be applied also to cardiac or respiratory gated data, as reported by the authors of reference [2]. Instead of assuming a parameterised motion model, one can try to jointly estimate image and motion by minimising a cost function depending on the likelihood of the image and motion and a regularisation part depending on the smoothness and size of the deformation. This kind of approach has been attempted by many authors [3,4,5] and has its main drawback in the computational cost of the minimisation.

We apply, here, a method that uses reconstructed gates for estimating the deformation fields among them and then reconstructs the entire data set in a chosen reference gate by incorporating the deformation fields in the reconstruction algorithm. A similar approach was already proposed in 2006 by Qiao and co-authors [6] using CT-gated images for gate registration. Pouchol et al., in reference [7], instead, proposed to use PET gate registration by means of a deep-learning-based algorithm [7]. This approach has the advantages of not requiring an extra dose to the patient and to be computationally inexpensive enough to be adopted in clinical practice. We improve the results obtained in reference [7], by using SynthMorph [8] for the estimation of the registration fields. SynthMorph has the advantage of being able to estimate the deformation field at low computational cost compared to traditional methods and, most importantly, to be contrast and modality agnostic [9]. In other words, SynthMorph is capable of registering gates with substantially different numbers of events and can also register PET images to X-ray CT images (or Magnetic Resonance images).

There is a further complication in the reconstruction of the entire data set in a chosen reference gate that is due to the photon attenuation by the patient. PET scanners can only detect photons that exit the patient; in fact, a substantial amount of photons will be absorbed by the patient tissues and will depose a dose. In order to estimate the radiotracer distribution, projections, therefore, have to be corrected for this attenuation, which is strongly dependent on the specific tissues encountered by the emitted photons along the path of the different projection lines. For this reason, it is, at present, customary to perform an X-ray Computed Tomography (CT) in concomitance with a PET scan. The CT reconstruction is then used to estimate an attenuation map for attenuation correction. The challenge in the case of respiratory or cardiac gating resides in the fact that, in general, none of the emission gates will exactly correspond to the position in which the X-ray CT scan has been taken and the misalignment between the attenuation map and the various gates will introduce serious artefacts in the reconstruction.

In this paper, two algorithms for performing the appropriate attenuation correction of the data are proposed. In one of the proposed algorithms (referred to as M-MLEM with CT-based attenuation correction or, in short M-MLEM+CT in the following) a CT-image is scaled to PET photon energies (that is 511 keV) and registered to each of the gates via Synthmorph, whereas, in the second proposed approach (referred to as *M-MLAA* in the remainder of this paper), Synthmorph is composed with the forward projector in a morphed version of the Maximum Likelihood Activity and Attenuation (MLAA) [10] algorithm. Both of these proposed methods will be described in more detail in the next section.

## 2. Materials and Methods

In this paper, the algorithm proposed in reference [7] and reported for convenience in Algorithm 1, is extended to 3D and the attenuation correction of the projections is included in the reconstruction in two different ways.
**Algorithm 1** M-MLEM algorithm**Require:** 
n≥0
**Require:** 
m≥0
**Require:** 
N≥1  A←A                          ▹ Radon transform  $A^{*}\leftarrow A^{*}$                 ▹ adjoint operator of Radon transform  θ←θ               ▹ learned parameters of Synthmorph network  $n\leftarrow MLEM_{iter}$                      ▹ nb of MLEM iterations  $m\leftarrow MMLEM_{iter}$                  ▹ nb of M-MLEM iterations  $N\leftarrow nb_{gates}+1$                             ▹ nb total of gates  $f_{0},\ldots ,f_{N}\leftarrow 1\ldots 1$               ▹ init of the distribution estimation  $g_{0},\ldots ,g_{N}$                        ▹ data for every gates  $W_{0},\ldots ,W_{N}\leftarrow Id,\ldots ,Id$                ▹ Init registration fields  **for** 
i←0,N
**do**      **for** k←1,n **do**          $f_{i}\leftarrow f_{i}\frac{A^{*}g_{i}}{Af_{i}^{k}A^{*}1}$                        ▹ MLEM iteration      **end for**      **if** i>0 **then**          $W_{i,i-1}\leftarrow H\left(f_{i-1},f_{i},\theta \right)$         ▹ vector field between two close gates          $W_{i-1,i}\leftarrow -W_{i,i-1}$            ▹ approximate estimation of inverse          $W_{i}\leftarrow W_{i,i-1}\circ \ldots \circ W_{1,0}$          $W_{i}^{-1}\leftarrow W_{0,1}\circ \ldots \circ W_{i-1,i}$      **end if** **end for** **for**
k←1,m
**do**      $f_{0}\leftarrow \frac{\sum_{j=1}^{N}A_{j-1}^{*}\left[\frac{g_{j}}{A_{j}\left(f_{0}^{k}\right)}\right]}{\sum_{j=1}^{N}A_{j-1}^{*}1}$  $f_{0}\frac{\sum_{j=0}^{N}\left|\det D\varphi_{j}\right|\left(A^{T}\frac{g_{j}}{A(f_{0}\circ \varphi_{j}^{-1})}\right)\circ \varphi_{j}}{\sum_{j=0}^{N}\left|\det D\varphi_{j}\right|\left(A^{T}1\right)\circ \varphi_{j}}$               ▹ M-MLEM iteration **end for**


### 2.1. Proposed Algorithms: M-MLEM with CT-Based Attenuation Correction and M-MLAA

Let us consider the case of gated sinogram data with N+1 gates $g_{0},\ldots ,g_{N}$, in each of which the patient is assumed to be stationary. The corresponding images of the activity distribution are going to be denoted by $f_{0},\ldots ,f_{N}$ and the relative attenuation maps by $\mu_{0},\ldots ,\mu_{N}$, whereas the attenuation map obtained from an X-ray CT scan will be indicated by $\mu_{CT}$.

Data acquisition is modelled by a *forward operator*, *A*, such that:

(1) $$Af_{j}e^{-A\mu_{j}}=g_{j}.$$

It is here worth noting that Equation (1) refers to the expectation values of $f_{j}$ and $g_{j}$, which are both, in fact, Poisson-distributed random variables.

Finally, let $W_{j}$ be the registration action between gates $f_{j}$ and $f_{j+1}$:

$$f_{j}=W_{j}f_{j+1}$$

and let us define $A_{j}$ as the composition of the forward operator with the registration action: $A_{j}\equiv A\circ W_{j}$, so that $g_{j}=A_{j}f_{j+1}e^{-A\mu_{j+1}}$.

Disregarding the effect of attenuation, the reconstruction of all data in the reference gate, $f_{0}$, can be performed using the Morphed-Maximum Likelihood Expectation Maximisation, M-MLEM, introduced in reference [7] and reported in Algorithm 1.

#### 2.1.1. M-MLEM with CT-Based Attenuation Correction

The first algorithm proposed in this paper performs the attenuation correction by deforming the CT-based attenuation map, $\mu_{CT}$, to the reference gate and will be called the Morphed-Maximum Likelihood Expectation Maximisation with CT-based attenuation map (*MLEM + CT*) in the remainder of this work. The activity distribution is updated as reported in Algorithm 2, where $\mu_{CT-0}$ denotes the CT-based attenuation map after it has been morphed to the reference gate.
**Algorithm 2** M-MLEM + CT algorithm**Require:** 
n≥0

**Require:** 
m≥0
**Require:** 
p≥0
**Require:** 
N≥1  A←A                          ▹ Radon transform  $A^{*}\leftarrow A^{*}$                 ▹ adjoint operator of Radon transform  θ←θ               ▹ learned parameters of Synthmorph network  $m\leftarrow MLAA_{iter}$                     ▹ nb of MLAA iterations  $p\leftarrow MMLEM_{iter}$                   ▹ nb of M-MLEM iterations  $N\leftarrow nb_{gates}+1$                             ▹ nb total of gates  $f_{0},\ldots ,f_{N}\leftarrow 1\ldots 1$                ▹ init of the distribution estimation  $g_{0},\ldots ,g_{N}$                        ▹ data for every gates  $W_{0},\ldots ,W_{N}\leftarrow Id,\ldots ,Id$                 ▹ init registration fields  **for** 
k←0,m
 **do**  $f_{0}^{k+1}\leftarrow f_{0}^{k}\frac{A^{*}\left[e^{-A(\mu_{0}^{k})}\frac{g_{0}}{\left[e^{-A(\mu_{0}^{k})}A\left(f_{0}^{k}\right)\right]}\right]}{A^{*}\left[1\cdot e^{-A(\mu_{0}^{k})}\right]}$        ▹ MLAA iteration - MLEM step  $\mu_{0}^{k+1}\leftarrow \mu_{0}^{k}+\frac{A^{*}\left[e^{-A(\mu_{0}^{k})}A\left(f_{0}^{k}\right)\right]}{A^{*}\left[e^{-A(\mu_{0}^{k})}\cdot A\left(1\right)\right]}$           ▹ MLAA iteration - MLTR step  $W_{CT,0}\leftarrow H\left(\mu_{0},\mu_{CT},\theta \right)$    ▹ vector field between CT attenuation map and $\mu_{0}$  $W_{0,CT}\leftarrow -W_{CT,0}$             ▹ approximate estimation of inverse  $\mu_{CT-0}\leftarrow W_{CT,0}\left(\mu_{0}\right)$           ▹ attenuation map deformed in gate0  **for** 
i←0,N
**do**          **for** k←1,n **do**              $f_{i}^{k+1}\leftarrow f_{i}^{k}\frac{A^{*}\left[e^{-A(\mu_{i}^{k})}\frac{g_{i}}{\left[e^{-A(\mu_{i}^{k})}A\left(f_{i}^{k}\right)\right]}\right]}{A^{*}\left[1\cdot e^{-A(\mu_{i}^{k})}\right]}$      ▹ MLAA iteration: MLEM step              $\mu_{i}^{k+1}\leftarrow \mu_{i}^{k}+\frac{A^{*}\left[e^{-A(\mu_{i}^{k})}A\left(f_{i}^{k}\right)\right]}{A^{*}\left[e^{-A(\mu_{i}^{k})}\cdot A\left(1\right)\right]}$        ▹ MLAA iteration: MLTR step          **end for**          **if** i>0 **then**              $W_{i,i-1}\leftarrow H\left(f_{i-1},f_{i},\theta \right)$        ▹ vector field between two close gates              $W_{i,i-1}\leftarrow H\left(\mu_{i-1},\mu_{i},\theta \right)$        ▹ vector field between two close gates              $W_{i-1,i}\leftarrow -W_{i,i-1}$           ▹ approximate estimation of inverse              $W_{i}\leftarrow W_{i,i-1}\circ \ldots \circ W_{1,0}$              $W_{i}^{-1}\leftarrow W_{0,1}\circ \ldots \circ W_{i-1,i}$        **  end if** **end for**
  
**for** 
k←1,p
**do**      $f_{0}\leftarrow f_{0}^{k}\frac{\sum_{j=1}^{N}A_{j-1}^{*}\left[e^{-A_{j}\left(\mu_{CT-0}\right)}\frac{g_{j}}{e^{-A_{j}\left(\mu_{CT-0}\right)}A_{j}\left(f_{0}^{k}\right)}\right]}{\sum_{j=1}^{N}A_{j-1}^{*}\left[1\cdot e^{-A_{j}\left(\mu_{CT-0}\right)}\right]}$    $f_{0}\frac{\sum_{j=0}^{N}\left|\det D\varphi_{j}\right|\left(A^{T}\frac{g_{j}}{A(f_{0}\circ \varphi_{j}^{-1})}\right)\circ \varphi_{j}}{\sum_{j=0}^{N}\left|\det D\varphi_{j}\right|\left(A^{T}1\right)\circ \varphi_{j}}$             ▹ M-MLEM iteration  **end for**


#### 2.1.2. M-MLAA

The second proposed algorithm will be called the Morphed-Maximum Likelihood Activity and Attenuation (*M-MLAA*). It follows the interleaved updates of activity distribution and attenuation map as in its original version [10], but the forward and backward operators are composed with the registration fields evaluated by Synthmorph, as described in Section 2.1. The two update steps will be, consequently, called *M-MLEM* and *M-MLTR*, and are reported in Algorithm 3.
**Algorithm 3** M-MLAA**Require:** 
n≥0
**Require:** 
m≥0
**Require:** 
N≥1  A←A                          ▹ Radon transform  $A^{*}\leftarrow A^{*}$                 ▹ adjoint operator of Radon transform  θ←θ               ▹ learned parameters of Synthmorph network  $m\leftarrow MLAA_{iter}$                     ▹ nb of MLAA iterations  $p\leftarrow MMLEM_{iter}$                   ▹ nb of M-MLAA iterations  $N\leftarrow nb_{gates}+1$                             ▹ nb total of gates  $f_{0},\ldots ,f_{N}\leftarrow 1\ldots 1$                ▹ init of the distribution estimation  $g_{0},\ldots ,g_{N}$                        ▹ data for every gates  $W_{0},\ldots ,W_{N}\leftarrow Id,\ldots ,Id$                  ▹ init registration fields  **for** 
i←0,N
**do**      **for** k←1,n **do**          $f_{i}^{k+1}\leftarrow f_{i}^{k}\frac{A^{*}\left[e^{-A(\mu_{i}^{k})}\frac{g_{i}}{\left[e^{-A(\mu_{i}^{k})}A\left(f_{i}^{k}\right)\right]}\right]}{A^{*}\left[1\cdot e^{-A(\mu_{i}^{k})}\right]}$       ▹ MLAA iteration: MLEM step          $\mu_{i}^{k+1}\leftarrow \mu_{i}^{k}+\frac{A^{*}\left[e^{-A(\mu_{i}^{k})}A\left(f_{i}^{k}\right)\right]}{A^{*}\left[e^{-A(\mu_{i}^{k})}\cdot A\left(1\right)\right]}$         ▹ MLAA iteration: MLTR step      **end for**      **if** i>0 **then**          $W_{i,i-1}\leftarrow H\left(f_{i-1},f_{i},\theta \right)$         ▹ vector field between two close gates          $W_{i,i-1}\leftarrow H\left(\mu_{i-1},\mu_{i},\theta \right)$        ▹ vector field between two close gates          $W_{i-1,i}\leftarrow -W_{i,i-1}$            ▹ approximate estimation of inverse          $W_{i}\leftarrow W_{i,i-1}\circ \ldots \circ W_{1,0}$          $W_{i}^{-1}\leftarrow W_{0,1}\circ \ldots \circ W_{i-1,i}$      **end if**   **end for**
  **for** 
k←1,m 
**do**      $f_{0}\leftarrow f_{0}^{k}\frac{\sum_{j=1}^{N}A_{j-1}^{*}\left[e^{-A_{j}\left(\mu_{CT-0}\right)}\frac{g_{j}}{e^{-A_{j}\left(\mu_{CT-0}\right)}A_{j}\left(f_{0}^{k}\right)}\right]}{\sum_{j=1}^{N}A_{j-1}^{*}\left[1\cdot e^{-A_{j}\left(\mu_{CT-0}\right)}\right]}$    $f_{0}\frac{\sum_{j=0}^{N}\left|\det D\varphi_{j}\right|\left(A^{T}\frac{g_{j}}{A(f_{0}\circ \varphi_{j}^{-1})}\right)\circ \varphi_{j}}{\sum_{j=0}^{N}\left|\det D\varphi_{j}\right|\left(A^{T}1\right)\circ \varphi_{j}}$        ▹ MMLAA iteration: MMLEM step      $\mu_{0}^{k+1}\leftarrow \mu_{0}^{k}+\frac{\sum_{j=1}^{N}A_{j-1}^{*}\left[e^{-A_{j}\left(\mu_{0}^{k}\right)}A_{j}\left(f_{0}^{k}\right)\right]}{\sum_{j=1}^{N}A_{j-1}^{*}\left[e^{-A_{j}\left(\mu_{0}^{k}\right)}\cdot A_{j}\left(1\right)\right]}$      ▹ MMLAA iteration: MMLTR step  **end for**


### 2.2. Implementation of the Algorithms

Forward and backward operators are implemented from the Time of Flight (TOF) branch of the Synergistic Image Reconstruction Framework (SIRF) library [11], and are wrapped in the Operator Discretization Library (ODL) [12] in which all the algorithms are implemented. The operators are defined according to the geometry of the Siemens mCT scanner.

Registration fields are computed using the pre-trained convolutional neural network Synthmorph [8]. Synthmorph is trained on synthetic, semantic segmented images and provides modality-agnostic and contrast-invariant registration. Registration fields, $W_{j}$, registering $f_{j}$, and $f_{j+1}$ are computed from images obtained with two initial MLAA iterations.

Inverse diffeomorphisms, $W_{j}^{-1}$, are computed by the first-order approximation of $W_{j}$, as: $-W_{j}\left(\cdot \right)$. For both approaches, two sets of diffeomorphisms for the activity distributions and the attenuation map are evaluated. In order to enhance the Synthmorph registration performances, the attenuation map reconstructions are segmented to remove the artifacts in the region of the field of view not covered by the object. These artifacts are commonly seen whenever applying an MLTR-MLAA update [13]. For *M-MLEM + CT*, Synthmorph is further deployed as a first step in the registration of the CT-based attenuation map, $\mu_{CT}$, to the reconstruction of the reference gate, $f_{0},$ obtained after the same number of initial MLAA iterations.

### 2.3. Synthetic Data Generation

In order to assess the performance of the proposed algorithms, synthetic projection data have been generated from the digital 4D extended cardiac-torso XCAT phantom [14] in which five lesions have been added.

The voxel size of the generated XCAT phantom is 0.316 × 0.316 × 0.16 cm${}^{3}$ and four respiratory and cardiac gates have been used in this study. Please note that the proximity to the real respiratory and cardiac motion of the XCAT phantoms is ensured by the fact that they were obtained from X-ray CT and Magnetic Resonance measurements of healthy male volunteers. In particular, 4D-tagged Magnetic Resonance Imaging data and 4D high-resolution respiratory-gated CT data are used [14].

The size, relative uptake, and positioning of the lesions have been carefully chosen. The size is almost the same for all of the lesions and comparable to the system resolution.

**Figure 1:**
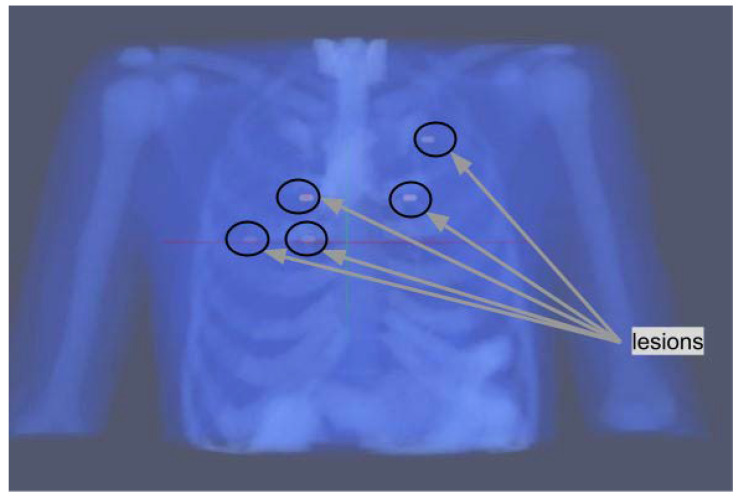
Position of the five lung lesions in the XCAT phantoms.

Their position is shown in Figure 1; two lesions are localized in slice 55, two lesions in slice 65, and one lesion in slice 80. The rationale for their positioning is described in the following. One of the lesions is placed in the upper part of the lungs, this should be the easiest one to detect as respiratory motion is lower in that region. Two of them are placed in proximity to the heart, which has an high uptake through the blood pool, and are therefore expected to have a lower contrast compared to their surroundings. The remaining two lesions are placed around the middle of the lungs, one on each side.

Uptake is chosen to be either 4:1 or 6:1 compared to the blood pool, reflecting the typical standard uptake values in fluorodeoxyglucose PET.

From the labels in the XCAT phantom, a radiotracer distribution and an attenuation map have been estimated in the way described in the following:

**Radiotracer distribution:** Uptake for healthy tissues has been taken from reference [15] with an educated guess for tissues not present in the reference. The activity in reference [15] has been scaled to a level consistent to the dose injected at Karolinska Hospital in Huddinge (3 MBq/kg up to a max of 400 MBq total). The scaling has been done considering a mass of 70 kg for the full-body XCAT-phantom and by setting the average uptake per voxel to be equal to the total injected dose divided by the number of voxels in the XCAT-phantom. Lungs uptake is considered to be 0.75 of the average uptake and the scaling factors for remaining organs are derived from Table 2 in reference [15] by dividing the median activity concentration in the organ (column 2) by the median activity of lungs divided by 0.75. The five lesions were given an activity corresponding to 4 or 6 times that of the blood pool. One of the lesions in slice 55 has a dimension of 6 × 5 voxels, whereas the other is 6 × 4 and the lesions in slice 80 and 65 are all 6 × 5 voxels in dimension.
**Attenuation map:** The attenuation map has been estimated from the labels of the XCAT using the values from the National Institute of Standards and Technology tables [16]. Since, at the energy of interest, most human tissues, apart from bone and lung tissues, have similar attenuation, we only used the x different value for the linear attenuation coefficients reported in Table 1.

Once the activity distribution and the attenuation map have been created, attenuated sinograms are generated with forward projection and Poisson noise is added.

### 2.4. Hardware and Software

The hardware used is an Intel(R) Xeon(R) CPU E5-2690 v3 @ 2.60 GHz with one GPU from the ASPEED Graphics Family, with 500 Gigabyte memory and 8 Terabyte hard disk. All computations are run in Python 3.7.4, using TOF capabilities of the Synergistic Image Reconstruction Framework (SIRF 3.4) library and the Operator Discretization Library (ODL 0.7.0). Voxelmorph 0.2 and Tensorflow 2.11.0 are used for Synthmorph registration.

### 2.5. Evaluation of M-MLAA and M-MLEM + CT Reconstructions

*M-MLAA* and *M-MLEM + CT* reconstructions have been compared against:

- MLAA reconstruction of reference gate, $f_{0}$; this corresponds to the situation in which a large amount of projection data are thrown away in order to avoid motion blurring (called, in the remainder of this study, *Single-gate MLAA*);
- MLAA reconstruction from the sum of the data in all of the four gates; this corresponds to disregarding the motion correction (called, in the remainder of this study, *Total-gate MLAA*);
- MLAA single-gate reconstructions summed after registration in the reference gate with Synthmorph (called, in the remainder of this study, *Sum + Synthmorph*);
- MLEM reconstruction of the reference gate, $f_{0}$, with attenuation correction performed through a CT-derived attenuation map. This corresponds to the most common clinical practice and disregards motion correction (called, in the remainder of this study, *Clinical Standard MLEM*).

The comparison of the algorithm performances is carried out through the evaluation of chosen Figures of Merit (FOMs) and a Channelised Hotelling Observer (CHO).

#### 2.5.1. Figures of Merit (FOMs)

The FOMs that have been evaluated are: Peak Signal-to-Noise Ratio (PSNR), Recovery Coefficient (RC), and Signal Difference-to-Noise Ratio (SDNR). PSNR has been evaluated, on the whole, using reconstructed images with a data range equal to 500; RC and SDNR have been evaluated within Regions of Interest (i.e., ROIs) around the five lesions in the phantom.

With $I_{1}$ indicating the phantom, $I_{2}$ the reconstructed image from noisy and attenuated sinograms, $\Omega_{l}$ the ROI around the lesion, and $\Omega_{b}$ the ROI within the background, the chosen FOMs have been evaluated as follows:

- PSNR:
  (2) $$PSNR=10log_{10}\left(\frac{d^{2}}{MSE}\right)$$
  Here, *d* is the maximum fluctuation in the input image data type and MSE is the Mean Squared Error, computed as follows:
  (3) $$MSE=\sum_{M,N}\frac{{\left[I_{1}\left(m,n\right)-I_{2}\left(m,n\right)\right]}^{2}}{M\cdot N}$$
- RC:
  (4) $$RC=\frac{\sum_{\Omega_{l}}I_{2}}{\sum_{\Omega_{l}}I_{1}}$$
- SDNR:
  (5) $$SDNR=\frac{\frac{1}{N_{pixel_{l}}}\sum_{\Omega_{l}}I_{2}-\frac{1}{N_{pixel_{b}}}\sum_{\Omega_{b}}I_{1}}{\sqrt{\sigma \left(I_{1}\left(\Omega_{l}\right)\right)+\sigma \left(I_{1}\left(\Omega_{b}\right)\right)}}$$

#### 2.5.2. Channelised Hotelling Observer

Model observers are used to assess the quality of medical images with respect to a clinical task of interest, which, in our case, is a lesion detection task. They function as a powerful surrogate of human performance [17,18]. The Channelised Hotelling Observer, CHO, belongs to the class of linear model observers, which act by applying a linear template w to the image data vector f to compute a scalar test statistic *t* as a decision variable, according to the following:

(6) $$t=w^{T}f$$

The decision variable is, then, compared against a threshold to determine if the lesion is present (t>threshold) or absent (t<threshold). By varying the threshold, it is possible to compute the Receiver Operating Characteristic (ROC) Curve and the Area Under the Curve (AUC) and use those to compare the performances of the tested algorithms [18,19]. The CHO operates through a set of channels, which allow for extracting specific features from the images, such as the ones that can be extracted by the human visual system. The outputs of the channels are then linearly combined using the *Fisher–Hotelling rule* in order to achieve the optimal CHO template. The Fisher–Hotelling rule states that the vector a, containing the best linear combination of the channel weights, is to be computed as follows [20]:

(7) $$a={K_{v}}^{-1}\left[<f_{V/s}>-<f_{V/b}>\right]$$

Here, ${K_{v}}^{-1}$ is the covariance matrix of the output of the channels to the images, $<f_{V/s}>$ is the mean signal plus background, as seen by each channel, and $<f_{V/b}>$ is the mean background only, as seen by each channel. The optimal template is, finally, used to compute the test statistic and evaluate the ROC and AUC for each of the algorithms tested.

In this paper, the CHO is implemented through 4 Gabor filters. Gabor filters have been chosen since they are a validated model to mimic the response of the cells in the visual cortex [19,20]. The parameters of the Gabor filters have been set to a spatial frequency that varies between 0.01 and 0.02 pixels, an orientation that varies between 0.01 and 0.05 radians, a bandwidth of 0.9 octaves, and with standard deviation set to 3. Twenty-five ROIs have been considered, each with a dimension of 10×10 pixels. Five ROIs are evaluated around the lesions of interest and twenty in the background only; they are taken within the slices 55, 65, and 80. Before feeding in the CHO and extracting features through the channels, a Gaussian filter with σ=0.8 is used to reduce the noise within the reconstructed images.

## 3. Results

### 3.1. Visual Inspection

**Figure 2:**
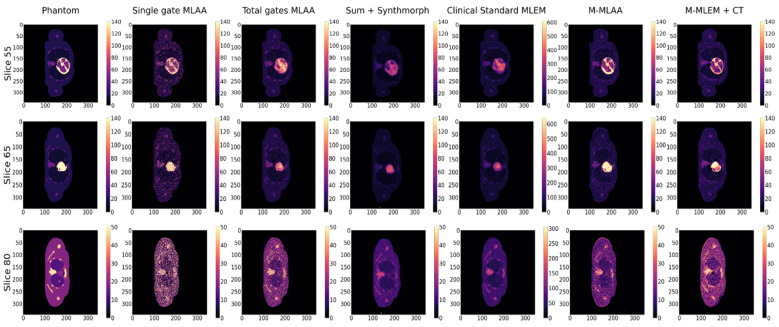
Slices 55, 65, and 80 of the XCAT phantom (uttermost left column) and their reconstructions with the different algorithms compared in this work.

**Figure 3:**
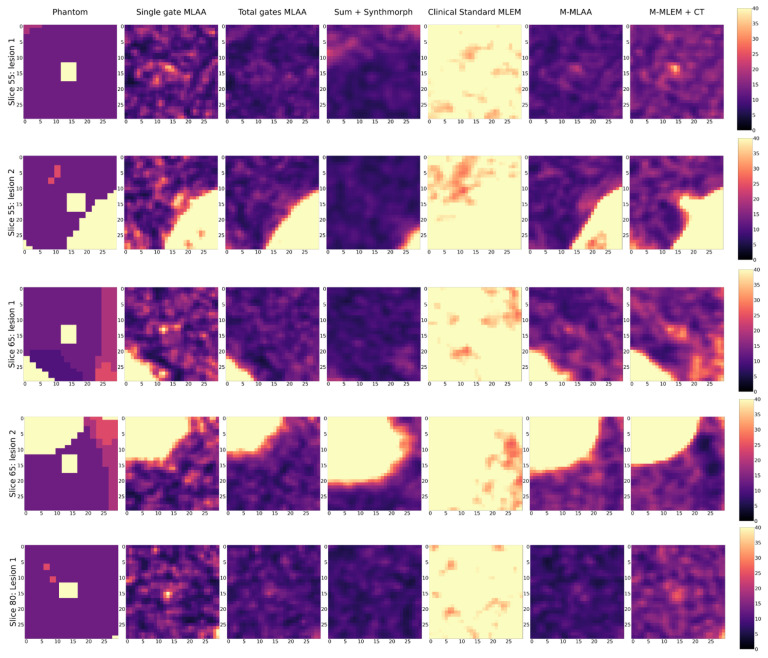
Blow-up of a region of interest around the five lung lesions in the XCAT phantom (uttermost left column) and of the same region of interests in the image reconstructed with the different algorithms compared in this work.

For the purpose of visual inspection, the reconstruction of the entire slices (i.e., 55, 65, and 80) containing the lesions, and the reconstructions of a region of interest around the lesions (with a dimension of 25 × 25 pixels) are reported, for each of the algorithms tested, in Figure 2 and Figure 3, respectively.

The number of iterations has been fixed to 50 for all the algorithms, except for *Clinical Standard MLEM*, which is instead visualised after 20 iterations and at the same dynamic range as the other algorithms. This choice is dictated by the fact that *Clinical Standard MLEM* is commonly regularised by early stopping, since it is known that the *MLEM* reconstructions tend to become noisier after a certain number of iterations [21]. Thus, we have shown the reconstructions from *Clinical Standard MLEM* at the best of the algorithm performance, according to the qualitative assessment of the visibility of the small lesions.

In Figure 3, a blow-up of the area around the lesions in the different reconstructions is shown. A Gaussian filter with σ = 0.8 has been applied to reduce the noise of the reconstructions. A visual estimate of the contrast and spatial resolution of the reconstructions, obtained using the different tested algorithms in the area surrounding the lesions, suggests that *Single gate MLAA*, *M-MLAA*, and *M-MLEM + CT* are the best-performing algorithms.

Please note that the *Clinical Standard MLEM* reconstruction of the activity obtained after 20 iterations has a much higher value than the actual value, as well as than any of the other reconstructions. We have chosen to present the reconstruction with the same dynamic range as the others in order to make the comparison somewhat fair.

By a visual inspection of Figure 2 and Figure 3, we can make different observations regarding the reconstruction of the five lesions. The two lesions in slice 55 are better visually reconstructed in *M-MLEM + CT* and *M-MLAA*, with single-gateMLAA being highly noisy and easily leading to the misinterpretation of the artefacts. Blurring from motion can be clearly observed in ClinicalStandardMLEM and totalgatesMLAA.

In slice 65, the lesion that is very close to the heart is not captured by any of the tested algorithms, probably due to the combination of the high uptake in the heart and the cardiac movement. The second lesion in slice 65 seems to be reconstructed better by *M-MLAA* and *M-MLEM + CT*, which both provide better contrast and spatial resolution.

The lesion in slice 80 seems, similarly, to be captured better by the *M-MLEM + CT* algorithm.

**Figure 4:**
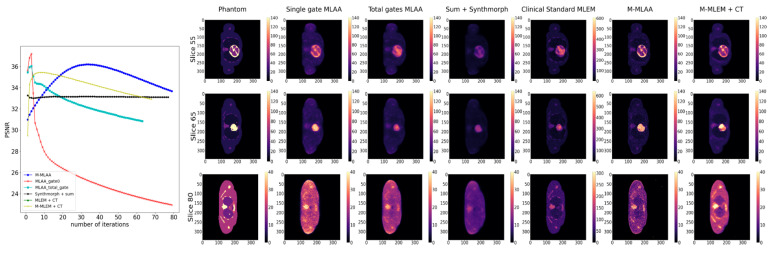
PSNR as a function of iteration number (left pane) and reconstruction of chosen slices at the optimal number of iterations for each of the tested algorithms.

**Figure 5:**
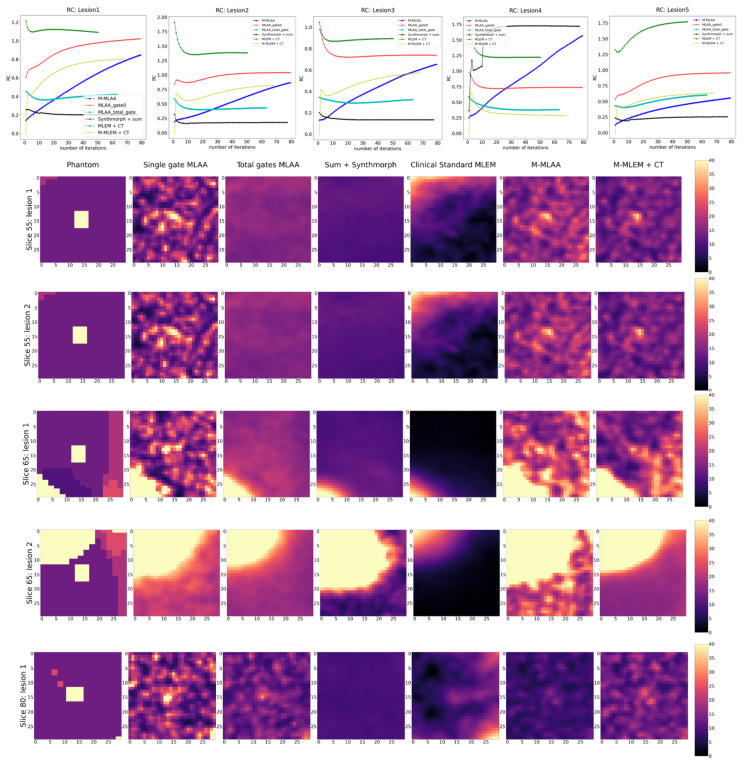
RC as a function of iteration number (top row) and reconstruction of the lesions at the optimal number of iterations for each of the tested algorithms.

**Figure 6:**
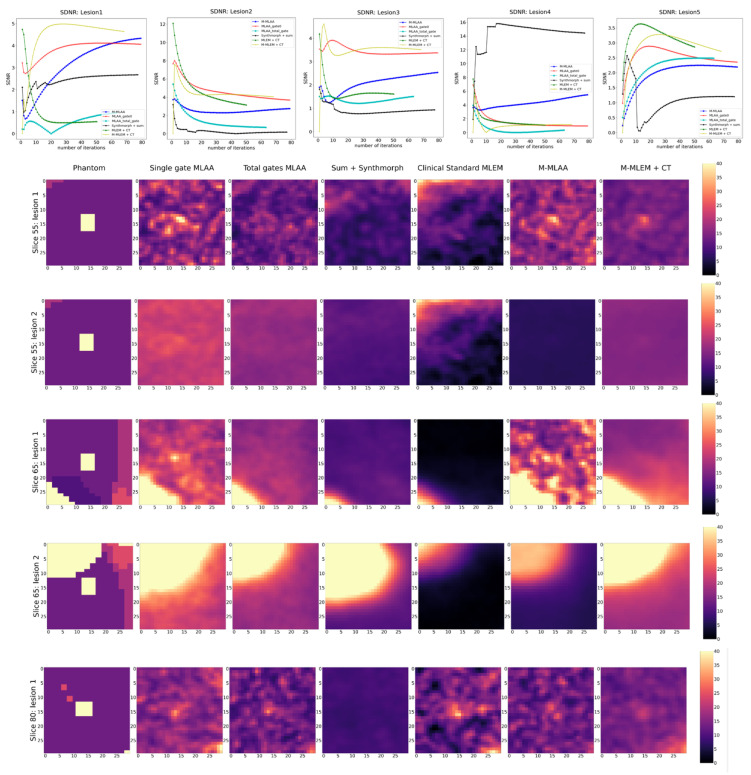
SDNR as a function of iteration number (top row) and reconstruction of the lesions at the optimal number of iterations for each of the tested algorithms.

In the following sections, instead, we show reconstructions at the iteration number that maximize the chosen figure of merit (see Figure 4, Figure 5 and Figure 6).

### 3.2. FOMs and CHO Results

In Figure 4, Figure 5 and Figure 6, the trends of the FOMs with respect to the number of iterations for each of the tested algorithms are plotted. This allows for determining the optimal number of iterations at which the FOM reaches the maximum value for each of the algorithms under investigation. Entire slices or only the lesions are, afterwards, reconstructed at each of the identified optimal numbers of iterations and reported correspondingly.

**Figure 7:**
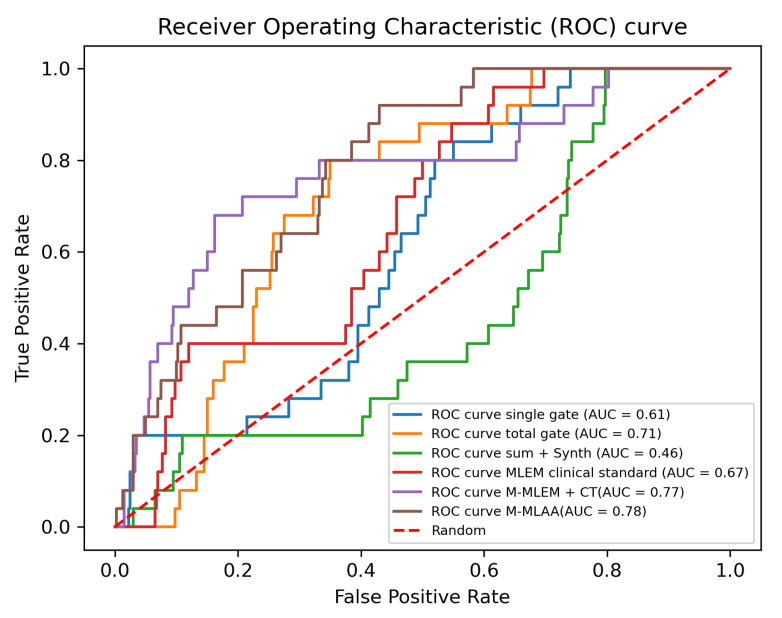
CHO implementation: ROC and AUC evaluation.

Finally, to further assess the quality of the reconstructed images, a CHO is implemented as described in Section 2. In order to compare the performances of the algorithms, ROC and AUC are plotted for each one of them and reported in Figure 7.

## 4. Discussion

Looking at Figure 4, Figure 5 and Figure 6, it is evident that the response from the chosen FOMs does not correlate with the visual assessment of the lesion visibility and fidelity to the phantom. In Figure 4, it is possible to observe that the maximum PSNR values are obtained by the *single-gate MLAA* after only a few iterations. However, the corresponding image seems to be of inferior quality compared to both *M-MLEM + CT* and *M-MLAA* at their best PSNR value, which is obtained for a larger number of iterations.

Similarly, in Figure 5, the RC value for each of the algorithms as a function of the number of iterations is shown together with the corresponding reconstructions of an ROI around the lesions at the optimal number of iterations. For all lesions, RC favours single-gateMLAA and total-gateMLAA. However, this does not seems to be the case when looking at the corresponding reconstructions.

Finally, SDNR shows rather inconsistent behaviour over the different lesions, making it difficult to establish which algorithm offers the best performance in reconstructing the lesions.

The results obtained with the CHO are, instead, more compatible with what the visual inspection suggests (see Figure 7), with *M-MLEM + CT* and *M-MLAA* outperforming the other algorithms and having similar performances (AUC of 0.78 and of 0.77, respectively).

According to the CHO, single-gateMLAA and sum+Synthmorph give the worst reconstructions, with sum+Synthmorph exhibiting a worse performance than simple random guessing. On passing, let us here point out that we are aware of the fact that, in cases like the one here presented, it is always possible to improve the performance of an observer that is performing under the 0.5 AUC threshold (corresponding to random guessing), by simply reversing the observer predictions. This is, however, not relevant in the present case as the above inversion would still yield an AUC value of 0.54, which is still close to random guessing.

As a last note, it is worth noting that scatter and random corrections have not being considered in this study and need to be included before applying the two proposed algorithms to clinical data. This does not constitute a substantial challenge, since the algorithms can be easily extended to account for those two corrections, just as is routinely done with MLEM. Also, the hyperparameters of both *M-MLEM + CT* and *M-MLAA* can be further optimised, in particular regarding the optimal number of initial MLAA iterations before feeding reconstructed images in Synthmorph for the evaluation of the registration fields.

## 5. Conclusions and Future Perspectives

The *M-MLEM* algorithm presented in reference [7] has been further developed to enable the reconstruction of the entire 3D volume from a realistic PET scan at once, and attenuation correction has been added to the algorithm. Our results on synthetic data suggest that the proposed *M-MLEM + CT* and *M-MLAA* algorithms have the potential to improve PET imaging diagnostic power for small pulmonary lesions at a low extra computational cost compared to standard MLEM or MLAA. Considering that SynthMorph does not need any training on clinical data, there is a good potential for this method to be clinically viable.

## Figures and Tables

**Table 1:** Linear attenuation coefficients used for obtaining an attenuation map at 511 keV from the XCAT phantom labels.

Tissue/Material	Linear Attenuation Coefficient (cm${}^{-1}$)
air	0
lung tissue	0.01
bladder	0.05
liver	0.08
cartiladge	0.15
bone	0.17
remaining tissues	0.1

## Data Availability

Not applicable.

## References

[1] Reader A.J., Verhaeghe J. (2014). 4D image reconstruction for emission tomography. Phys. Med. Biol..

[2] Rahmim A., Tang J., Zaidi H. (2013). Four-dimensional image reconstruction strategies in cardiac-gated and respiratory-gated PET imaging. PET Clin..

[3] Jacobson M., Fessler J.A. Joint estimation of image and deformation parameters in motion-corrected PET. Proceedings of the 2003 IEEE Nuclear Science Symposium. Conference Record (IEEE Cat. No. 03CH37515).

[4] Jacobson M.W., Fessier J. Joint estimation of respiratory motion and activity in 4D PET using CT side information. Proceedings of the 3rd IEEE International Symposium on Biomedical Imaging: Nano to Macro.

[5] Blume M., Martinez-Moller A., Keil A., Navab N., Rafecas M. (2010). Joint reconstruction of image and motion in gated positron emission tomography. IEEE Trans. Med. Imaging.

[6] Qiao F., Pan T., Clark J.W., Mawlawi O.R. (2006). A motion-incorporated reconstruction method for gated PET studies. Phys. Med. Biol..

[7] Öktem O., Pouchol C., Verdier O. (2019). Spatiotemporal PET Reconstruction Using ML-EM with Learned Diffeomorphic Deformation. Machine Learning for Medical Image Reconstruction.

[8] Hoffmann M., Billot B., Greve D.N., Iglesias J.E., Fischl B., Dalca A.V. (2022). SynthMorph: Learning Contrast-Invariant Registration Without Acquired Images. IEEE Trans. Med. Imaging.

[9] Hinkle J., Szegedi M., Wang B., Salter B., Joshi S. (2012). 4D CT image reconstruction with diffeomorphic motion model. Med. Image Anal..

[10] Rezaei A., Defrise M., Bal G., Michel C., Conti M., Watson C., Nuyts J. (2012). Simultaneous reconstruction of activity and attenuation in time-of-flight PET. IEEE Trans. Med. Imaging.

[11] Ovtchinnikov E., Brown R., Kolbitsch C., Pasca E., da Costa-Luis C., Gillman A.G., Thomas B.A., Efthimiou N., Mayer J., Wadhwa P. (2020). SIRF: Synergistic image reconstruction framework. Comput. Phys. Commun..

[12] Adler J., Kohr H., Öktem O. (2017). Operator Discretization Library. https://github.com/odlgroup/odl.

[13] Cheng L., Ma T., Zhang X., Peng Q., Liu Y., Qi J. (2020). Maximum Likelihood Activity and Attenuation estimation using both emission and transmission data with application to utilization of Lu-176 background radiation in TOF PET. Med. Phys..

[14] Segars W.P., Sturgeon G., Mendonca S., Grimes J., Tsui B.M. (2010). 4D XCAT phantom for multimodality imaging research. Med. Phys..

[15] Vauclin S., Michel C., Buvat I., Doyeux K., Edet-Sanson A., Vera P., Gardin I., Hapdey S. (2015). Monte-Carlo simulations of clinically realistic respiratory gated 18 F-FDG PET: Application to lesion detectability and volume measurements. Comput. Methods Programs Biomed..

[16] Hubbell J.H., Seltzer S.M. (1995). Tables of X-ray mass attenuation coefficients and mass energy-absorption coefficients 1 keV to 20 MeV for elements Z = 1 to 92 and 48 additional substances of dosimetric interest. Technical Report.

[17] Zhang L., Cavaro-Ménard C., Le Callet P. (2014). An overview of model observers. IRBM.

[18] He X., Park S. (2013). Model observers in medical imaging research. Theranostics.

[19] Eckstein M.P., Bartroff J.L., Abbey C.K., Whiting J.S., Bochud F.O. (2003). Automated computer evaluation and optimization of image compression of X-ray coronary angiograms for signal known exactly detection tasks. Opt. Express.

[20] Calvo Gonzalez M. (2011). Channelized Hotelling Observer Optimization for Medical Image Quality Assessment in Lesion Detection Tasks. Master’s Thesis.

[21] Gaitanis A., Kontaxakis G., Spyrou G., Panayiotakis G., Tzanakos G. (2010). PET image reconstruction: A stopping rule for the MLEM algorithm based on properties of the updating coefficients. Comput. Med. Imaging Graph..

